# Development of Generic Immuno-Magnetic Bead-Based Enzyme-Linked Immunoassay for Ustiloxins in Rice Coupled with Enrichment

**DOI:** 10.3390/toxins13120907

**Published:** 2021-12-17

**Authors:** Yi Huang, Xiaoqian Tang, Lu Zheng, Junbin Huang, Qi Zhang, Hao Liu

**Affiliations:** 1 The Key Lab of Plant Pathology of Hubei Province, Huazhong Agricultural University, Wuhan 430070, China; hzauhuangyi@163.com (Y.H.); luzheng@mail.hzau.edu.cn (L.Z.); junbinhuang@mail.hzau.edu.cn (J.H.); 2 Oil Crops Research Institute, Chinese Academy of Agricultural Sciences, Wuhan 430062, China; wtxqtutu@163.com

**Keywords:** rice false smut, ustiloxins, generic antigen, immuno-magnetic beads, enzyme-linked immunity

## Abstract

Ustiloxins are a group of mycotoxins produced by rice false smut pathogen. Previous studies have shown that the false smut balls contain six types of ustiloxins, and these toxins are toxic to living organisms. Thus, immunoassay for on-site monitoring of ustiloxins in rice is urgently required. The current immunoassays are only for detecting single ustiloxin, and they cannot meet the demand for synchronous and rapid detection of the group toxins. Therefore, this study designed and synthesized a generic antigen with ustiloxin G as material based on the common structure of the mycotoxins. Ustiloxin G was conjugated to two carrier proteins including bovine serum albumin (BSA) and ovalbvmin (OVA) by carbon diimide method. The mice were immunized with ustiloxin-G-BSA to generate the antibody serum, which was further purified to obtain the generic antibody against ustiloxins. The conjugated ustiloxin G-OVA and generic antibodies were used for establishing the enzyme-linked immunosorbent assay (ELISA) for ustiloxin detection and optimizing experiment conditions. The characterization of the antibody showed that the semi-inhibitory concentrations (IC_50_) of ustiloxin A, B, and G were 0.53, 0.34, and 0.06 µg/mL, respectively, and that their corresponding cross-reactivities were 11.9%, 18.4%, and 100%, respectively. To increase ELISA detection efficiency, generic antibody was combined with magnetic beads to obtain sensitive and class-specific immune-magnetic beads. Based on these immuno-magnetic beads, a high-efficiency enzyme-linked immunoassay method was developed for ustiloxin detection, whose sensitivity to ustiloxin A, B, and G was improved to 0.15 µg/mL, 0.14 µg/mL, and 0.04 µg/mL, respectively. The method accuracy was evaluated by spiking ustiloxin G as standard, and the spiked samples were tested by the immune-magnetic bead-based ELISA. The result showed the ustiloxin G recoveries ranged from 101.9% to 116.4% and were accepted by a standard HPLC method, indicating that our developed method would be promising for on-site monitoring of ustiloxins in rice.

## 1. Introduction

Rice false smut (RFS) has become an emerging and economically important fungal disease in most rice-producing countries recently [1,2,3,4,5,6]. Since the 1970s, the harm of RFS has been widely reported in the major planting areas in China. After the 1980s, with the change of farming methods and the large-scale promotion of hybrid rice varieties, RFS has exhibited an increasing trend year by year, and currently, it harms nearly one third of the rice cultivation area [7,8,9]. The damage of RFS has been constantly investigated. In addition to causing the obvious yield loss, it can also produce large amounts of toxins to pollute grains [10,11,12,13]. Of all the known toxins produced by RFS pathogen, ustiloxins are the most widely concerned.

Ustiloxins are a group of cyclopeptides containing a 13-membered cyclic core structure with an ether linkage. To date, six types of ustiloxins have been identified and named as ustiloxin A, B, C, D, F, and G, respectively [11,14,15,16]. Among them, ustiloxin A and B are the main species, accounting for about 90% of the total content of ustiloxins in mature false smut balls (FSBs) [12]. Numerous studies have revealed that ustiloxins are widely toxic to animals. When fed with contaminated rice grains or feeds for a short time, the domestic animals show a variety of symptoms mainly including diarrhea, hemorrhage, vomiting, ovarian atrophy, and abortion [17,18]. Both ustiloxin A and the crude water extract of FSBs have been reported to cause acute necrosis of liver and kidney in mice [10,14,19,20]. The latest toxicology studies show that ustiloxin A can affect development of early-stage zebrafish [21] and may reduce the population of *Tetrahymena thermophila* by disrupting the cell cycle [22]. Since RFS is becoming increasingly serious in China, ustiloxin pollution has posed a great threat for food safety.

Detection techniques toward mycotoxins are important for the safety of agricultural products. At present, the main detection methods of mycotoxins include instrumental analysis detection and immunoanalysis detection. Instrumental analysis is mainly applied in laboratories, industries, and farmers’ enterprises. It is always accurate and reliable, but tends to require expensive instruments and highly-skilled professionals. In recent years, the research on rapid immune detection of small molecule targets has developed rapidly, and immunoassays have been widely used for on-site rapid detection in food security field. The mechanisms of antibody affinity and stability have been newly interpreted [23,24]. Many new antibodies have been developed, such as nanoantibodies and molecular engineering antibodies [25,26]. A number of rapid detection devices and corresponding analysis methods have emerged such as optical and electrochemical immune sensors, new immune kits, and test paper strips [27,28,29]. 

To the best of our knowledge, research on the rapid detection technology of ustiloxins is still preliminary. A high-performance liquid chromatographic method for determing ustiloxin A was described, and the limit of detection was 2.5 mg/kg [30]. Then, a HPLC-MS method was developed for simultaneous detection of multiple ustiloxins. The detection limit of ustiloxin A was 5.5 mg/g, while for total ustiloxins it was 0.2 mg/g [31]. In addition to the conventional instrumental detection, the monoclonal antibody enzyme-linked immunoassays against ustiloxin A and B were also reported [32,33]. However, the available monoclonal antibody immunoassays and the corresponding products only target single ustiloxin, thus it is hard to meet the current requirement for simultaneous rapid screening of multiple toxins. Considering this, it is urgent to develop a general rapid detection technology. In the current work, generic hapten against ustiloxins was synthesized and conjugated to protein to produce generic immunogen, which was further injected into mice to generate generic antiserum. The magnetic bead-antibody complex was prepared by immobilizating the general antibody on the surface of magnetic beads. Based on the magnetic bead-antibody complex, an improved ELISA method for the rapid detection of group ustiloxins was established. Our method has great potential to be applied for multi-residue detection of ustiloxins.

## 2. Results and Discussion

### 2.1. Design, Synthesis, and Identification of Generic Antigen

**Figure 1:**
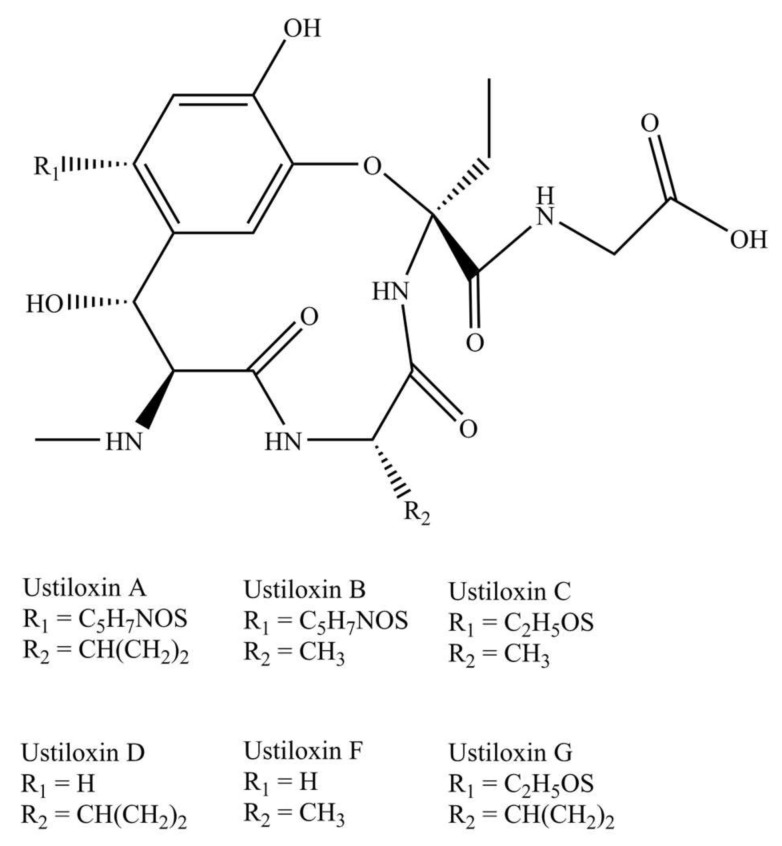
Chemical structures of ustiloxin A, B, C, D, F, and G.

Ustiloxins are a group of cyclopep-tides containing a 13-membered cyclic core structure with an ether linkage. The chemical structures of the six known ustiloxins are shown in Figure 1.

As for the antigen design, we mainly considered the following three design principles in reference to the previously reported method: (1) The group exposed to antigen should be the common moieties, which contributes to high affinity interaction between hapten and antibody; (2) the group exposed to antigen should exhibit similar polarity among analytes; (3) steric hindrance of the substituents should be as small as possible [34]. 

It should be emphasized that phenyl, amino, and hydroxyl are the molecular basis for the formation of high-affinity interaction between hapten and antibody. According to the above design principle and the structures of the six ustiloxins, the exposure of the common phenyl, amino, and hydroxyl groups of the ustiloxins to the surface of the carrier protein will be conducive to the production of class-specific antibodies. Therefore, we chose the carboxyl to couple with carrier protein so as to promote the exposure of these groups. Additionally, considering the polar similarity of substituents (Residue 1 and Residue 2 in Figure 1) and other factors, we finally chose ustiloxin G as the raw material for antigen synthesis.

**Figure 2:**
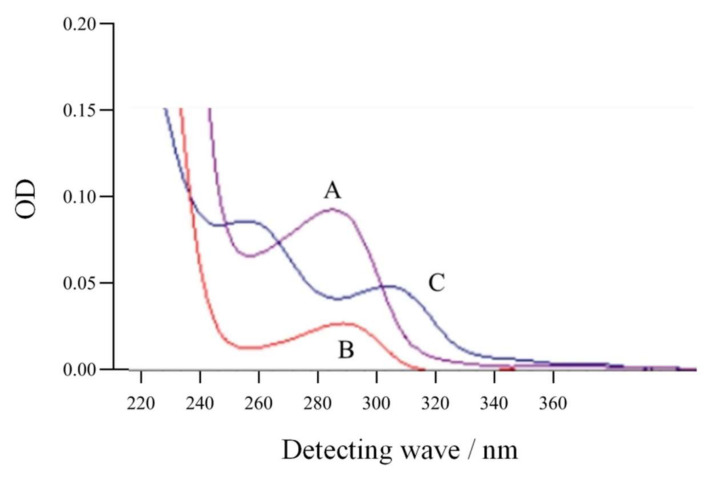
Ultraviolet scanning spectra of ustiloxin G (A), bovine serum albumin (B), and the generic antigen of ustiloxins (C).

The generic antigen was synthesized through active ester method, and the small molecules that did not bind to the carrier proteins were removed. The generic antigen was identified by ultraviolet scanning method. The scanning spectra of the synthesized antigen, the raw material ustiloxin G, and carrier protein BSA (Figure 2) showed that the antigen had two characteristic absorption peaks at 254 nm and 291 nm, that ustiloxin G had a unique absorption peak at 275 nm, and that BSA had a unique absorption peak at 280 nm. These results indicated that the conjugation reaction of the artificial antigen (ustiloxin G-BSA) was successful. Following the same procedures, ustiloxin G-OVA was prepared as a coating antigen.

### 2.2. Preparation and Characterization of Generic Antibodies against Ustiloxins 

**Figure 3:**
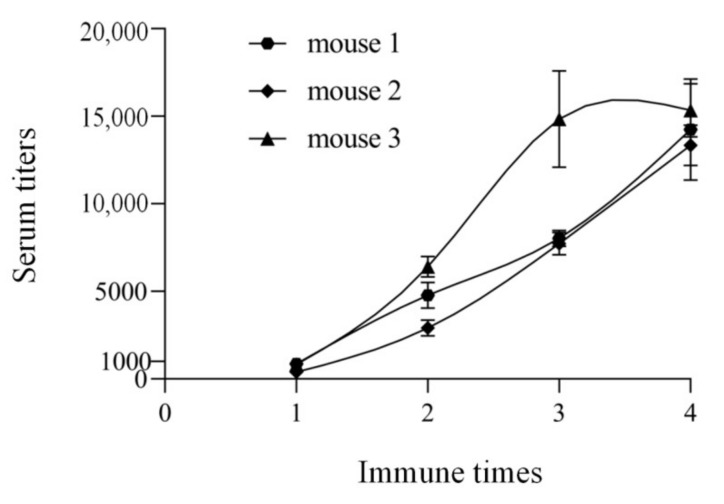
Titer trends of three antisera against generic antigen of ustiloxins.

In order to evaluate the above antigen, three mice were immunized with the generic antigen ustiloxin G-BSA by a three-point immune methods. The antisera were collected on the 7th day after each immunization, and the titers were determined by indirect non-competitive ELISA. The titer was expressed as the dilution multiple when the OD value was 1.0. The trends of the titers of the three antisera showed that the polyclonal antibody from mouse 3 had the highest titer (Figure 3). Thus, this polyclonal antibody was selected for the subsequent research. 

Using an optimized indirect competitive ELISA, the antibody’s sensitivity to ustiloxins was determined, and the result (Table 1) showed that IC_50_ was 0.53 µg/mL for ustiloxin A, 0.34 µg/mL for ustiloxin B, and 0.06 µg/mL for ustiloxin G. The cross-reactivity was 18.4%, 11.9%, and 100% for ustiloxin A, B, and G, respectively. 

### 2.3. Establishment of Class-Specific Immuno-Magnetic Bead Enzyme-Linked Immunoassay 

**Figure 4:**
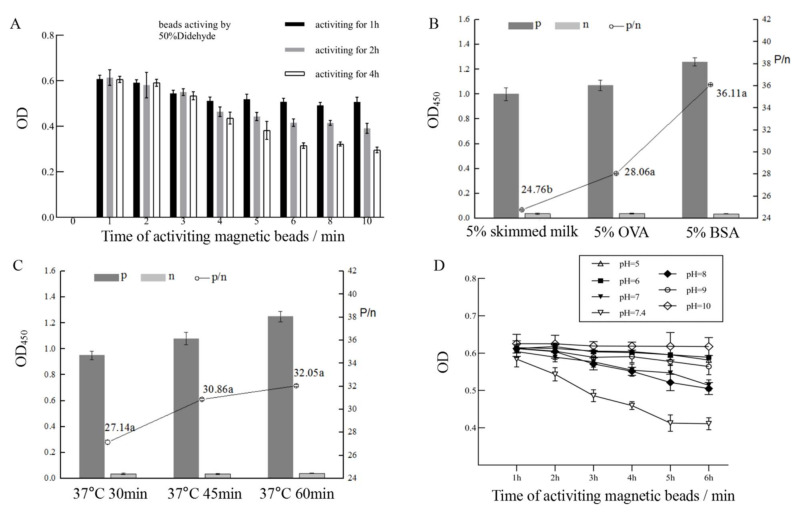
Optimization of immuno-magnetic bead-based enzyme-linked immunoassay conditions including the time length of activation of magnetic beads by glutaraldehyde and the time length of bead-antibody coupling reaction post magnetic activation (**A**), blocking regent types (**B**), antibody reaction time length in ELISA (**C**), and pH values (**D**).

In order to develop an immuno-magnetic bead enzyme-linked immunoassay, we firstly investigated the time length of magnetic bead activation by glutaraldehyde and the time length of bead-antibody coupling reaction post the bead activation. The shortest coupling time was determined once the OD value reached the maximum. The results indicated that when the magnetic beads were activated by glutaraldehyde for 4 h and then reacted with antibody for 10 min, the OD showed the highest value (Figure 4A). Under this condition, the immuno-magnetic beads of ustiloxins were prepared for further study. According to a classic chessboard titration method [35], the appropriate concentrations of the above immuno-magnetic beads were calculated as 2.0 μg/mL in terms of antibody concentration and that of the coated antigen (ustiloxin G-OVA) was determined as 2.5 μg/mL. 

Several factors can affect antibody-antigen binding reaction such as blocking regent types, pH value, and reaction time length. Compared with 5% skimmed milk and 5% OVA, 5% BSA as a blocking reagent was the best selection to prevent nonspecific binding (Figure 4B). Sixty minutes at 37 °C was found to be the optimal reaction time length (Figure 4C). The pH value experiment showed that about 7.4 was the most suitable pH value (Figure 4D). 

**Figure 5:**
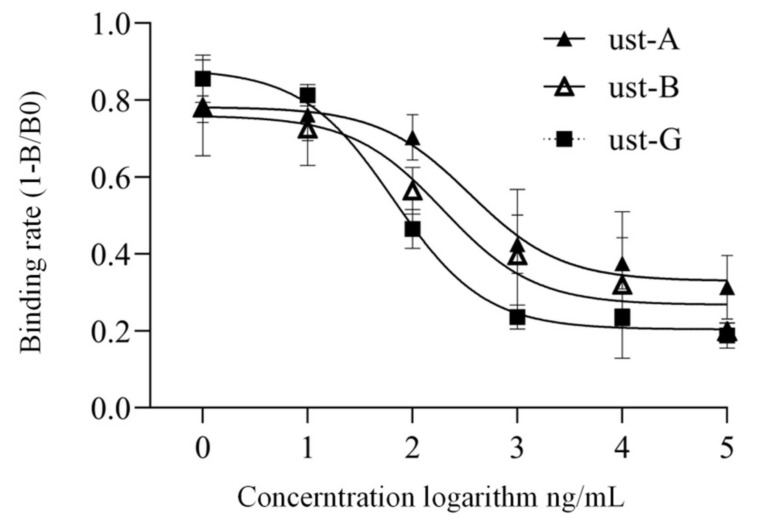
Immuno-magnetic bead-based enzyme-linked immunoassay curves for ustiloxin A, B, and G.

Finally, based on the above optimized conditions, a generic immuno-magnetic bead enzyme-linked immunoassay method was established for ustiloxin detection. The results indicated that the sensitivity of the established method for ustiloxin A, B, and G was 0.15 µg/mL, 0.14 µg/mL, and 0.04 µg/mL, respectively (Figure 5). It is worth mentioning that our immuno-magnetic bead-based ELISA method obviously improved sensitivity to ustiloxins with the cross-reactivity of ustiloxin A increased to 27.4% and that of ustiloxin B increased to 29.1%.

### 2.4. Evaluation of Accuracy and Standard Deviation 

In order to evaluate the accuracy and standard deviation of the developed method above, clean rice was spiked with ustiloxin G as standard. The result (Table 2) showed that the average ustiloxin G recoveries at the high-, mid-, and low-spiked concentration in rice were 101.9%, 107.4%, and 116.4% respectively. These concentration results of our developed method were very close to the theoretical concentrations. As shown in Table 2, the relative standard deviations of our developed ELISA were below 15%, indicating a good repeatability.

We further validated our developed ELISA by using a standard HPLC method and found that the results obtained from both methods were very similar, indicating that the tested results from immuno-magnetic bead-based ELISA could be accepted by the standard HPLC method. Additionally, our result also indicated that the sensitivity of our developed immunoassay was higher than that of standard HPLC. 

## 3. Conclusions

**Figure 6:**
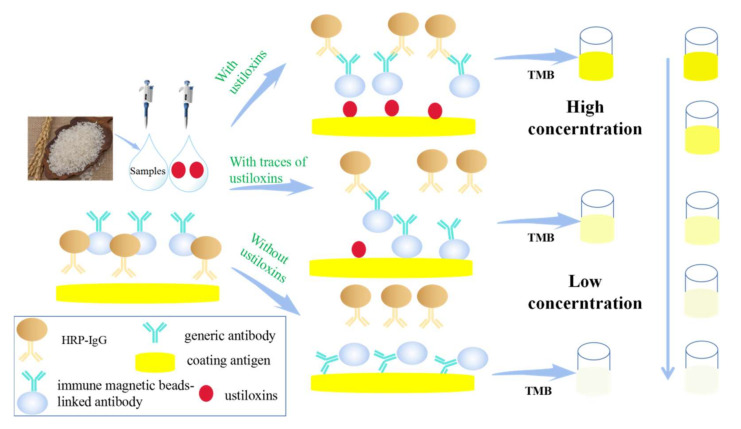
Generic immuno-magnetic bead-based enzyme-linked immunoassay for ustiloxins.

In view of the lack of universal rapid screening methods for ustiloxins in rice, we synthesized a generic antigen BSA-ustiloxin G and a coating antigen OVA-ustiloxin. With the conjugate of BSA-ustiloxin G as an immunogen, a generic polyclonal antibody against ustiloxins was obtained and characterized. The result revealed that the generic antigen was suitable for the preparation of generic antibodies against ustiloxins. With the generic antigen and antibody, a sensitive and class-specific immuno-magnetic bead-based enzyme-linked immunoassay was developed for ustiloxin detection, and its sensitivity to ustiloxin A was 0.15 µg/mL, to ustiloxin B was 0.14 µg/mL, and to ustiloxin G was 0.04 µg/mL. The accuracy and repeatability evaluation showed that the recoveries of our developed method ranged from 101.9% to 116.4%, and the new method test result could be accepted by a standard HPLC method. Notably, the sensitivity of the developed method was higher than the standard HPLC, and cross-reactivity of our developed ELISA higher than that of conventional ELISA. Here, we provided a new, sensitive, and generic immunoassay, which can be used for on-site monitoring ustiloxins in rice. The schematic of the assay procedure is shown in Figure 6.

## 4. Materials and Methods

### 4.1. Instruments and Experimental Equipment

Costar-clear 96-well culture plates were purchased from Costar (New York, NY, USA). Wellwash 4MK2 automatic plate washer (Thermo, Waltham, MA, USA) and SpectraMax M2e phosphase marker were from Molecular Instruments (Molecules, Los Angeles, CA, USA). Ultrasonic Cleaner (Jiangsu KH-500E) was from Kunshan Hehuang Ultrasonic Instrument Co., Ltd. (Kunshan, China). Shimadzu Prominence LC-20AT high performance liquid chromatography system (Kyoto, Japan) consisted of two LC-20AT solvent delivery units, one SIL-20A autosampler, one SPD-M20A photodiode array detector, one CBM-20Alite systemcontroller, and one Synergi reversed-phase Hydro-C18 column (250 mm, 4.6 mm, 10 mm) (Phenomenex, Torrance, CA, USA).

### 4.2. Materials and Reagents

The mycotoxins ustiloxin A (UST-A), ustiloxin B (UST-B), and ustiloxin G (UST-G) used in this study were prepared in our laboratory, according to the previously reported method [34,35]. 1-ethyl-(3-dimethylaminopropyl)-carbodiimide hydrochloride (EDC), N-hydroxysuccinimide (NHS), Freund’s incomplete adjuvant (FIA, Freund’s incomplete adjuvant), Freund’s complete adjuvant (FCA), bovine serum albumin (BSA), ovalbumin (OVA), 3, 3′, 5, 5′-tetramethylbenzidine (TMB), standards of aflatoxin B1 (AFB1), deoxynivalenol (DON), zearalenone (ZEA), and ochratoxin (OTA) were purchased from Sigma-Aldrich (St. Louis, MO, USA). Horseradish peroxidase-labeled goat anti-mouse IgG antibody (HRP-IgG) was purchased from Wuhan Boster Biological Co., Ltd. (Wuhan, China). Magnetic beads (700 nm, with –NH_2_) were purchased from Hangzhou Kunteng Nano Technology Co., Ltd. (Hangzhou, China).

Phosphate buffer (PBS) was prepared as follows. NaCl (16 g), KCl (0.4 g), Na_2_HPO_4_ 12H_2_O (5.8 g), and KH_2_PO_4_ (0.4 g) were added simultaneously into a volumetric flask, and then deionized water was added to reach a final volume of 2000 mL. The coating solution was formulated as follows. Na_2_CO_3_ (3.18 g) and NaHCO_3_ (5.86 g) were added together into a volumetric flask, and then deionized water was added to obtain carbonate buffer with a volume of 2000 mL and concentration of 0.05 mol/L. ELISA detergent was prepared as follows. One milliliter tween-20 was dissolved in 2 L PBS buffer (0.01 mol/L) and mixed well to obtain 0.05% PBST. Citric acid buffer was obtained by the following procedures. Na_2_HPO_4_·12H_2_O (9.205 g) and citric acid (4.665 g) were added into deionized water to obtain 500 mL solution, which was stored at 4 °C. Urea peroxide (3.0 g) was dissolved into 100 mL anhydrous ethanol to obtain urea peroxide solution, which was stored at 4 °C. The 0.2 g TMB was dissolved into 100 mL anhydrous ethanol to obtain TMB solution, and it was stored at −20 °C. The 0.5 mL TMB solution, 32 μL urea peroxide solution, and 9.5 mL citric acid buffer were mixed to obtain TMB color solution. The 11 mL concentrated sulfuric acid was dissolved into 89 mL deionized water to obtain stopping coloring solution, and it was stored at room temperature.

### 4.3. Preparation of Immunogen and Coating Antigen

The 1.0 mg Ustiloxin G was dissolved in 1.0 mL N,N-dimethylformamide (DMF), and then 2.485 mg N-hydroxysuccinimide (NHS) and 1.0 mg 1-ethyl-(3-dimethylaminopropyl)-carbodiimidehydrochloride (EDC) were added and mixed well. The mixture was placed at room temperature and stirred at 150 r/min for 2 h. The supernatant containing the active ester of ustiloxin G was finally obtained for subsequent experiments.

The 1.0 mg carrier protein (BSA or OVA) was dissolved in 1.0 mL PBS (0.01 mol/L), and then the active ester solution of ustiloxin G was added drop by drop into the protein solution when stirring. The mixture was then placed into a shaker at 4 °C and shaken at 200 r/min overnight. Finally, those small molecules unbound to protein were removed by ultrafiltration and centrifugation at 4000 g/min for 30 min, and the conjugates were retained in the ultrafiltration tube and were re-dissolved with PBS.

### 4.4. Preparation of Polyclonal Antibody against Ustiloxins

In the initial immunization, 2 mg USTG-BSA conjugate was dissolved in a sterilized 0.5 mL NaCl solution (0.9%) and then emulsified with an equal volume of FCA. Three 6-week-old female BALB/c mice were immunized by multiple-point subcutaneous injection with the final water-in-oil emulsion described above. Booster injections were performed with an equal volume of the FIA-emulsified antigen three times at 3-week intervals. To investigate the immune response to immunogen, the antisera were collected from the tails of the four mice at day 9 post immunization and assayed with ustiloxin G-OVA by indirect noncompetitive ELISA.

To examine the sensitivity and specificity of antibodies, the inhibition experiments of mycotoxin AFB1; DON; ZEA; OTA; and UST-A, B, and G were conducted using a traditional indirect competitive ELISA, and their 50% inhibition concentrations (IC_50_) were calculated by the classic four-parameter equation [27]. The specificity was usually expressed as cross-reactivity, which was calculated according to the following formula.

Cross-reactivity = (IC_50_ UST-G/IC_50_ UST-A, B, or other mycotoxin) ∗ 100%

Briefly, the procedures of the traditional indirect competitive ELISA were as follows: (1) The plates were coated with 100 mL/well USTG-OVA at an appropriate concentration in 0.05 M PBS (pH 7.4) and stood for 2 h at 37 °C (2). After washing three times with 300 mL 0.05% PBST, 200 mL of 5% OVA in the PBST solution was added to each well and incubated for 1 h at 37 °C. (3) After another three washes, the 100 mL/well generic polyclonal antibody against ustiloxins at an appropriate solution was added into each well of the plates; (4) after 1-h incubation at 37 °C, the plates were rewashed, IgG-HRP (diluted at 1/8000 in the PBST, 100 mL/well) was added, and then the plates were incubated for 45 min at 37 °C; (5). After six washes, the color was developed by adding 100 mL freshly prepared substrate solution (containing 9.5 mL phosphate-citrate buffer (pH 5.0) and 0.5 mL TMB (2 mg/mL) dissolved in ethanol), and 32 mL urea-hydrogen peroxide (3%, *w*/*v*)), and the mixture was incubated for 15 min at 37 °C in the dark; (6) the 50 mL of the stopping coloring solution (H_2_SO_4_) was added to each well, and the absorbance at 450 nm was measured with a microplate reader.

### 4.5. Preparation of Immuno-Magnetic Beads

The solvent of the magnetic bead solution (5 mg in 2 mL) was removed by magnetic separation, and magnetic beads were washed with 5 mL PBS solution (0.05 M) three times. Then 100 mL glutaraldehyde solution (50%) was added to the washed beads. The mixture was shaken for 4 h at room temperature.

After the activation, the magnetic beads were magnetically separated and washed three times with PBS solution. The beads were then added into the generic antibody solution (0.5 mg in 2.5 mL PBS) for conjugation for 10 min. The sites where the beads were unbound with the antibody were then blocked with 5% BSA for 1 h at room temperature. Finally, the conjugates were magnetically separated for subsequent use, and the residue antibodies in the reaction solution were tested by indirect noncompetitive ELISA.

### 4.6. Optimization of Generic Immuno-Magnetic Bead-Based Enzyme-Linked Immunoassay

OD value of indirect noncompetitive ELSA was used to evaluate immunoassay performance and to reveal the influence of multiple factors on reaction of the antigen–antibody complex. Multiple factors were investigated such as time length of the activation (1, 2, and 4 h) and the conjugation (1, 2, 3, 4, 5, 6, 8, and 10 min) between the immunomagnetic beads and antibody, blocking regent (5% OVA, 5% BSA, and 5% skim milk powder), time length of competitive reaction (30, 45, and 60 min) in the immuno-magnetic bead enzyme-linked immunoassay procedures, and pH (5.0, 6.0, 7.0, 7.4, 8.0, 9.0, and 10.0) of the competitive reaction solution.

It should be noted that the only difference between immuno-magnetic bead-based ELISA and the traditional ELISA lay in that the generic antibody was used directly for the traditional ELISA, whereas the immunomagnetic beads were used for the immuno-magnetic bead-based ELISA.

### 4.7. Evaluation on Accuracy and Repeatability of Usitloxin G Recovery

Three brands of clean rice uncontaminated by ustiloxin were collected from the local market. Ustiloxin G was used as the standard to evaluate the accuracy of the developed immunoassay. In the experiments, the uncontaminated rice was spiked with ustiloxin G, and then the spiked samples were ground. The ground samples were sonicated with ultrapure water for 4 h at room temperature. After centrifugation, the supernatant of each sample was filtered with a 0.22 μm microporous filter and transferred into a new tube before analysis. Theoretically, ustiloxin G at three final concentrations (10, 50, and 100 ng/mL) should be obtained. The extracts were finally detected by our developed immuno-magnetic bead enzyme immunoassay method and conventional HPLC method described previously [36,37].

## Figures and Tables

**Table 1:** Specificity of generic antibody against ustiloxins.

Analytes	IC_50_ (µg/mL)	Cross-Reactivity (%)
AFB1	no inhibition	0
DON	no inhibition	0
ZEN	no inhibition	0
OTA	no inhibition	0
UST-A	0.53	18.4
UST-B	0.34	11.9
UST-G	0.06	100

**Table 2:** Evaluation of developed method’s accuracy and standard deviation using spiked ustiloxin G as standards.

Method	Theoretical Concentration (ng/mL)	Tested Concentration (±SD)/(ng/mL)			Average Recovery (%)
		Rice Brand 1	Rice Brand 2	Rice Brand 3	
Immuno-magnetic bead enzyme immunoassay	0	no detection	no detection	no detection	no calculation
	10	10.96 ± 1.25	12.25 ± 0.92	11.70 ± 1.45	116.4
	50	50.14 ± 4.81	56.09 ± 5.25	54.93 ± 4.38	107.4
	100	101.36 ± 7.56	100.29 ± 8.92	104.00 ± 6.55	101.9
HPLC	10	no detection	no detection	no detection	no calculation
	50	no detection	no detection	no detection	no calculation
	100	105.49 ± 12.43	103.15 ± 7.92	105.81 ± 4.30	104.8
